# Supplementary material for: Formulation and validation of a regional household wealth index for sub-Saharan Africa
Source: PLoS One. 2025 Oct 31;20(10):e0335603. doi: 10.1371/journal.pone.0335603 (PMC12578161; doi:10.1371/journal.pone.0335603)
Supplement: S1 Table — (DOCX) [file pone.0335603.s001.docx]

# **Supporting Information**

**S1 Table. Movement of households from local to universal quintile with its associated tracking of funding reallocation and cost saving**

| Income Group | Country | Local 1st Quintile Covered (Poorest 20%) | Universal 1st Quintile Covered (Poorest 20%) | % Country Population in Local Quintile 1 | % Country Population in Universal Quintile 1 | Local-to-Universal Increase/Decrease | 2019 (GDP per capita, PPP | Average equivalent annual spend per CHW-2024 | Total Expenditure Original | Total Expenditure Universal | Universal $ - Local $ |
| --- | --- | --- | --- | --- | --- | --- | --- | --- | --- | --- | --- |
| Poorest 3rd | Mozambique | 3171 | 4760 | 10.3% | 15.5% | 33% | 1370 | $2,493 | $7,905,877 | $11,867,542 | $3,961,665 |
|  | Malawi | 1953 | 2662 | 6.4% | 8.7% | 27% | 1466 | $2,688 | $5,250,188 | $7,156,170 | $1,905,982 |
|  | Rwanda | 1249 | 2365 | 4.1% | 7.7% | 47% | 2325 | N/A | N/A | N/A | N/A |
|  | Uganda | 4446 | 5557 | 14.5% | 18.1% | 20% | 2444 | $624 | $2,773,449 | $3,466,499 | $693,050 |
|  | Lesotho | 224 | 84 | 0.7% | 0.3% | -167% | 2550 | N/A | N/A | N/A | N/A |
| Middle 3rd | Zimbabwe | 1553 | 1032 | 5.1% | 3.4% | -50% | 2712 | 1949 | $3,027,105 | $2,011,573 | -$1,015,532 |
|  | Tanzania | 5382 | 3268 | 17.6% | 10.7% | -65% | 2947 | 784 | $4,219,399 | $2,562,058 | -$1,657,341 |
|  | Zambia | 1960 | 3482 | 6.4% | 11.4% | 44% | 3386 | 1738 | $3,407,088 | $6,052,796 | $2,645,708 |
|  | Cameroon | 2413 | 1499 | 7.9% | 4.9% | -61% | 4196 | 4191 | $10,112,164 | $6,281,863 | -$3,830,302 |
| Richest 3rd | Kenya | 5021 | 4017 | 16.4% | 13.1% | -25% | 4711 | $716 | $3,595,735 | $2,876,731 | -$719,004 |
|  | Cote d’Ivoire | 2673 | 1749 | 8.7% | 5.7% | -53% | 5948 | $2,740 | $7,324,775 | $4,792,754 | -$2,532,021 |
|  | Eswatini | 119 | 36 | 0.4% | 0.1% | -231% | 9083 | $42,500 | $5,057,525 | $1,530,008 | -$3,527,517 |
|  | Namibia | 251 | 99 | 0.8% | 0.3% | -154% | 10410 | $16,783 | $4,212,553 | $1,661,525 | -$2,551,028 |
|  | Botswana | 240 | 45 | 0.8% | 0.1% | -433% | 14890 | $5,424 | $1,301,759 | $244,080 | -$1,057,680 |
|  | **Grand Total** | **30655** | **30655** | **100%** | **100%** |  |  |  | **$58,187,618** | **$50,503,598** | **-$7,684,020** |
|  |  |  |  |  |  |  |  |  |  | **Money Saved** | **-$7,684,020** |
|  |  |  |  |  |  |  |  |  |  | **% Saved** | **-15%** |
